# *Klebsiella pneumoniae* urinary tract infection: A multicentric study highlights significant regional variations in antimicrobial susceptibility across India

**DOI:** 10.1016/j.ijregi.2025.100605

**Published:** 2025-02-19

**Authors:** Meher Rizvi, Shalini Malhotra, Hiba Sami, Jyotsna Agarwal, Areena H. Siddiqui, Sheela Devi, Aruna Poojary, Bhaskar Thakuria, Isabella Princess, Aarti Gupta, Amal Al Malehi, Asfia Sultan, Ashish Jitendranath, Balvinder Mohan, Fatima Khan, Hatim El Tahir, Nainaraj Ilanchezhiyan, Mannu Jain, Maria Khan, Narendra Pal Singh, Renu Gur, Sarita Mohapatra, Shaika Farooq, Vellore Ramanathan Yamunadevi, Ken Masters, Nisha Goyal, Manodeep Sen, Razan Al Zadjali, Rugma Rajendradas, Suneeta Meena, Sudip Dutta, Bradley Langford, Reba Kanungo, Zaaima Al Jabri, Arwa Al Rajaibi, Sanjeev Singh, Azza Al Mamari, Sarman Singh, Keith H. St John, Raman Sardana, Pawan Kapoor, Amina Al Jardani, Rajeev Soman, Abdullah Balkhair, Neelam Taneja

**Affiliations:** 1 Department of Microbiology and Immunology, College of Medicine and Health Sciences, Sultan Qaboos University, Muscat, Oman; 2 Department of Microbiology, Atal Bihari Vajpayee Institute of Medical Sciences and Dr Ram Manohar Lohia Hospital, New Delhi, India; 3 Department of Microbiology, Jawaharlal Nehru Medical College and Hospital, Aligarh Muslim University, Aligarh, India; 4 Department of Microbiology, Dr Ram Manohar Lohia Institute of Medical Sciences, Lucknow, India; 5 Department of Microbiology, Integral Institute of Medical Science And Research, Lucknow, India; 6 Pondicherry Institute of Medical Sciences, Pondicherry, India; 7 Department of Pathology & Microbiology, Breach Candy Hospital Trust, Mumbai, India; 8 Microbiology, All India Institute of Medical Sciences Patna, Patna, India; 9 Apollo Speciality Hospitals, Vanagaram, Chennai, India; 10 Lab Operations, Agilus Diagnostics Limited, Fortis Memorial Research Institute, Gurugram, India; 11 College of Medicine and Health Sciences, Sultan Qaboos University, Muscat, Oman; 12 Department of Microbiology, Sree Gokulam Medical College and Research Foundation, Thiruvananthapuram; 13 Department of Medical Microbiology, Post Graduate Institute of Medical Education & Research, Chandigarh, Chandigarh, India; 14 Animal Health Research Centre, Ministry of Agriculture and Fisheries Wealth and Water Resources, Muscat, Oman; 15 Atal Bihari Vajpayee Institute of Medical Sciences and Dr Ram Manohar Lohia Hospital, New Delhi, India; 16 Microbiology Department, Surat Municipal Institute of Medical Education and Research (SMIMER), Surat, India; 17 Department of Pathology, Peshawar Institute of Cardiology-MTI, Peshawar, Pakistan; 18 Department of Microbiology, University College of Medical Sciences & GTB Hospital, New Delhi, India; 19 Department of Microbiology, Dr. Baba Saheb Ambedkar Medical College & Hospital, New Delhi, India; 20 Department of Microbiology, All India Institute of Medical Sciences, New Delhi, India; 21 Department of Microbiology, Government Meical College Srinagar, Srinagar, India; 22 Consultant Infection control, HOD CSSD & Operation Theatre Coordinator, Apollo hospitals, Chennai, India; 23 Medical Education and Informatics Department, College of Medicine and Health Sciences, Sultan Qaboos University, Muscat, Oman; 24 Department of Microbiology, University College of Medical Sciences and GTB Hospital, New Delhi, India; 25 Department of Biochemistry, College of Medicine and Health Sciences, Sultan Qaboos University, Muscat, Oman; 26 Department of Microbiology, Sree Gokulam Medical College and Research Foundation, Kerala, India; 27 Department of Laboratory Medicine, All India Institute of Medical Sciences, New Delhi, India; 28 University of Toronto, Toronto, Canada; 29 Department of Microbiology and Dean Research, Pondicherry Institute of Medical Sciences, Pondicherry, India; 30 Department of Medicine- Infection Diseases and Epidemiology, Amrita Institute of Medical Sciences, Amrita Vishwavidyapeetham, Faridabad, India; 31 Department of Microbiology, Al Masarra Hospital, Ministry of Health, Muscat, Oman; 32 Aarupadai Veedu Medical College and Hospital, Pondicherry, India; 33 North Star IPC Consulting Services, LLC, Pittsburgh, USA; 34 Clinical Microbiology and Infection Control, Indraprastha Apollo Hospitals, New Delhi, India; 35 Director, Board of Trustees, The International Federation of Integrated Care, Oxford, UK; 36 Honorary Secretary, Hospital Infection Society-India, New Delhi, India; 37 Chairman Steering Committee National Accreditation Board for Hospitals and Healthcare Providers, New Delhi, India; 38 Central Public Health Laboratories, Directorate General for Disease Surveillance and Control, Ministry of Health, Muscat, Oman; 39 Jupiter Hospital Pune, Pune, India; 40 Infectious Diseases Unit, Department of Medicine, Sultan Qaboos University Hospital, Sultan Qaboos University, Muscat, Oman

**Keywords:** *Klebsiella pneumoniae*, Uncomplicated urinary tract infections, Antimicrobial agents

## Abstract

**Objectives:**

Knowledge of local antibiotic susceptibility rates is essential to strengthen antimicrobial stewardship programs. *DASH to Protect Antibiotics* (https://dashuti.com/), promotes the dissemination of focused local antibiograms in community urinary tract infection (UTI). This study mapped the susceptibility profile of *Klebsiella pneumoniae* from 18 Indian centers.

**Methods:**

The centers spanned nine Indian States and three Union Territories. Urinary *K. pneumoniae* antibiograms from the outpatient clinic were collated and analyzed. Standardization was achieved through online training. For epidemiological purposes, five centers tested fosfomycin.

**Results:**

Overall, low susceptibility (<60%) was observed for the standard oral antibiotics prescribed for cystitis: co-trimoxazole, 54% (36-68%); ciprofloxacin, 52% (29-55%); amoxicillin-clavulanic acid, 46%, (35-82%); nitrofurantoin, 39% (19-78%); and cefuroxime, 30% (15-62%). The rates for third- and fourth-generation cephalosporins were 49% (45-52%) and 60% (36-76%), respectively. Extended-spectrum beta-lactamases ranged from 36-61% in India. Piperacillin-tazobactam 74% (65-87%), amikacin 76% (45-91%), and meropenem 81% (61-87%) exhibited higher activity. Fosfomycin was the most active antimicrobial, with 89% (92-97%) susceptibility. *K. pneumoniae* susceptibility was directly linked to geographic region, log gross domestic product (*P* <0.001), and humidity, and low and high temperatures (*P* <0.05).

**Conclusions:**

Oral treatment options for *K. pneumoniae* cystitis are rapidly decreasing. Northern and southern India showed significant differences in antimicrobial susceptibility, highlighting the importance of local antibiograms for promoting antimicrobial stewardship. For uncomplicated cystitis, co-trimoxazole (54%) may be the empirical choice. Good susceptibility to fosfomycin was observed (89%). The empirical use of fluoroquinolones, cephalosporins, and amoxicillin-clavulanic acid has been discouraged. Piperacillin-tazobactam and aminoglycosides are carbapenem-sparing agents.

## Highlights

• Mapping of the antimicrobial susceptibility of *Klebsiella pneumoniae* in urinary tract infection was undertaken by 18 centers across India.
• The national susceptibilities to nitrofurantoin, meropenem, and fosfomycin were 39%, 81%, and 89%, respectively.
• The proportion of susceptibility of *K. pneumoniae* to different antimicrobials was directly linked to geographic region (*P* <0.001).
• South India had the highest mean susceptibility to trimethoprim/sulfamethoxazole, piperacillin/tazobactam, imipenem, and meropenem.
• Humidity, and low and high temperatures (*P* <0.05) had a statistically significant effect on the proportion of susceptible *K. pneumoniae*.

## Introduction

*Klebsiella pneumoniae* is the second most common uropathogen associated with uncomplicated urinary tract infections (UTIs) [1,2]. Treating infections by *K. pneumoniae* is becoming increasingly challenging owing to the dramatic increase in antimicrobial resistance [3,4]. *K. pneumoniae* strains can acquire a variety of β-lactamase enzymes, which have the ability to disrupt the chemical structure of beta-lactam antibiotics, including the most extensively used antibiotics (penicillins, cephalosporins, and carbapenems) [5]. Although nitrofurantoin and fosfomycin are deemed feasible empirical treatment options for *Escherichia coli* cystitis, their suitability for *K. pneumoniae* is limited [6]. This substantially limits the choice of antimicrobials available for the management of such cases. A multicentric research group, DASH to Protect Antibiotics (https://dashuti.com), determines the most effective treatment options by analyzing the susceptibility profile of *K. pneumoniae* isolated from patients with community-acquired UTIs in India. Considering that the initial treatment for uncomplicated UTIs is almost always empirical, this exercise is vital for management. *K. pneumoniae* cystitis needs greater attention considering its inherent and increasing resistance to commonly used antimicrobial agents. In this study, antimicrobial susceptibilities were compared across five broad geographic regions: North, South, East, West, and Central India. Socioeconomic variables were studied to assess their impact on antimicrobial susceptibility.

## Materials and methods

**Figure 1:**
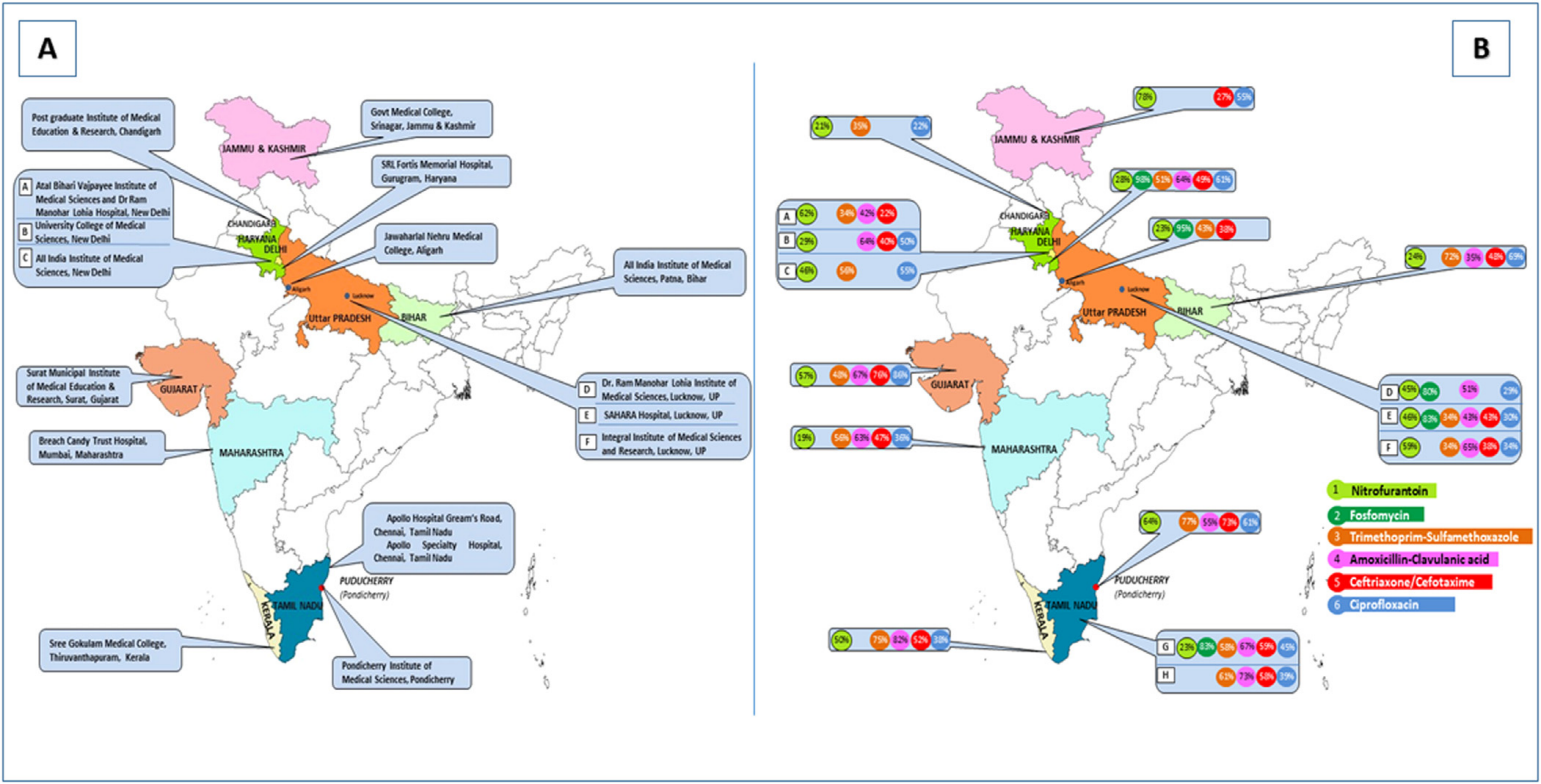
(a) and (b) Geographic location of participating centers and susceptibility profile of *Klebsiella pneumoniae* to commonly prescribed antimicrobials.

Eighteen centers participated in the study with their distribution being as follows: 11 in North (N.) India—one in the extreme North (Jammu & Kashmir), four in Delhi, one in the neighboring National Capital Region (NCR) Gurugram, one each in Aligarh and Chandigarh, three in Lucknow, four in South (S.) India (two in Chennai and one each in Pondicherry and Kerala), and two in West (W.) India (one each in Gujarat and Maharashtra), along with one center in the East (E.) (Patna, Bihar) (Figure 1). Owing to their proximity, Chandigarh (a Union Territory west of Delhi) and Gurugram (in Haryana but part of the NCR) were analyzed together with Delhi. The recruitment process has been outlined in a previous study [6]. The duration of this study was 1 year, from January 1, 2022 to December 31, 2022. Ethical approval for this study was obtained from the participating centers. The centers in N., C., and E. India are characterized by a subtropical climate; W. India is tropical, with defined wet and dry seasons, whereas S. India is tropical, with hot summers and dry winters.

### Processing of samples

Properly collected midstream urine samples from patients with symptoms were screened for bacteria and leucocytes by microscopy. Diagnosis was performed on the basis of the presenting symptoms and significant bacteriuria, i.e., ≥10^5^ colony forming units of organism on culture [7]. Among them, 12 centers used automated bacterial identification systems, and the remainder used manual methods. Antimicrobial susceptibility testing was performed according to Clinical and Laboratory Standards Institute (CLSI) guidelines (M100-Ed33) 2022 [8]. Ten sites largely used disk diffusion testing; 12 used automated systems, and six used a mixture of both approaches. Quality control was performed by all laboratories. Extended-spectrum beta-lactamases (ESBLs) were detected at eight centers using cephalosporin/clavulanic acid synergy tests.

CLSI urine breakpoints were used to interpret cefuroxime results. Isolates susceptible to dose-dependent breakpoints such as cefepime were deemed susceptible.

### Collection of data

Only clinical isolates from patients presenting with symptomatic UTI for the first time in the outpatient or emergency department were included. Data from such patients were collated into site antibiograms if 30 or more isolates were tested at the site. Only the data on routinely tested antimicrobial agents were included. The CLSI guideline M39A4E CLSI 2022 was used to prepare the antibiograms.

### Statistical analysis

The mean proportion of susceptibility to each antimicrobial was calculated on the basis of the mean (minimum, maximum) of different geographic regions of India. The means of the proportion of susceptibility were compared in geographic regions using the Kruskal-Wallis test. Two-way analysis of variance was performed to assess the simultaneous effect of the antimicrobial and geographic region on the proportion of susceptible *K. pneumoniae* while adjusting for other variables. The *P* significance was set at *P* <0.05. All statistical analyses were performed using SPSS version 29 software (IBM Corp., Armonk, NY, USA).

## Results

A total of 51,703 samples were obtained from the surveyed outpatient departments, of which 2649 (5%) contained community-acquired *K. pneumoniae*. Site-by-site details of the susceptibility rates of *K. pneumoniae* are listed in Table 1, and the regional rates for the major antibiotic groups are listed in Supplementary Table 1. The regional rates of standard urinary antibiotics are presented in Figure 1. Supplementary Figure 1 lists the regional variations in susceptibility to broad-spectrum antimicrobials. Antimicrobial susceptibilities were compared across four broad geographic regions: N., E., W., and S. India. Geographic locations, climatic variables, and socioeconomic variables are listed in Supplementary Table 2.

**Table 1:** Antimicrobial susceptibility profile of *Klebsiella pneumoniae* to the major antimicrobial groups.

The overall national susceptibility to nitrofurantoin was 39%, with very low rates in Mumbai 19%, Chandigarh 21%, Patna 24% and Chennai and Aligarh 23%. Across all five regions, fosfomycin was the most reliably active antimicrobial, with 89% (92-97%) susceptibility. The antimicrobial susceptibility to co-trimoxazole was low, ranging from 36-68%, with an average rate of 54%. Ciprofloxacin susceptibility was 52%, ranging from 29-55% in N. India, 36-86% in W. India, 38-61% in S. India, and 22-61% in Delhi.

Cefuroxime performed poorly, with only approximately 30% (15-62%) susceptibility nationwide. Among the third- and fourth-generation cephalosporins, susceptibility rates ranged between 45% and 52%, averaging 49%, whereas it was 60% for cefepime. ESBLs ranged from lowest in W. India (36%) to highest in N. India (61%) (Supplementary Figure 2).

Susceptibility to beta-lactam/beta-lactamase inhibitors varied. Overall, the susceptibility rates were 74% (65-87%) for piperacillin/tazobactam and 55% (35-82%) for amoxicillin/clavulanic acid; cefoperazone/sulbactam susceptibility was estimated to be 60% (45-82%). Higher susceptibility rates of 76% (45-91%) were observed for amikacin than for gentamicin, 69% (43-87%).

Nationwide susceptibility rates were 82% (62-90%) for imipenem and 81% (61-87%) for meropenem. Significantly higher rates of susceptibility to meropenem were observed in S. India (86%) and E. India (83%) than in other regions. Delhi had 76% susceptibility to carbapenems.

**Figure 2:**
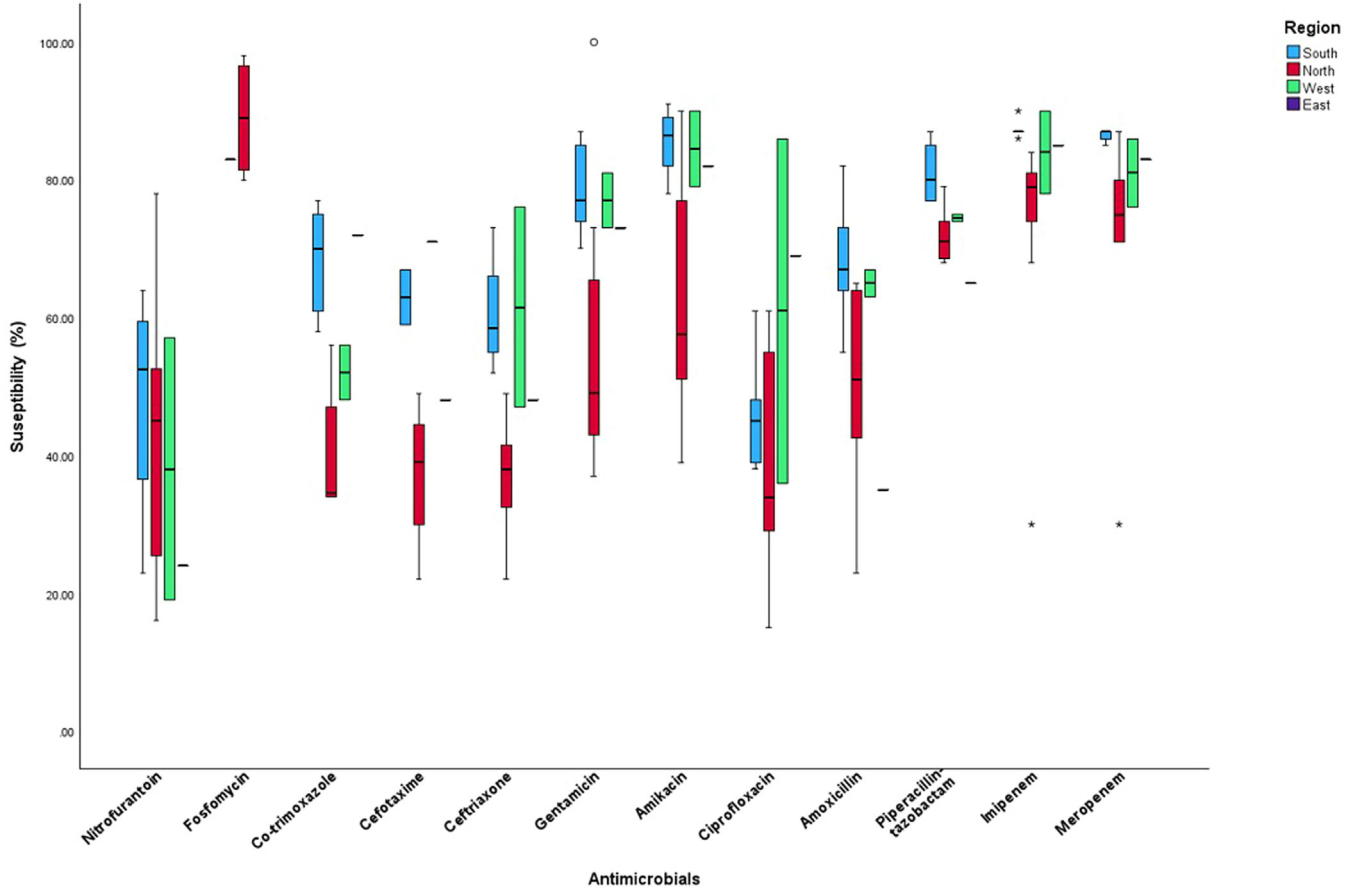
Regional variation in *K. pneumoniae* antimicrobial susceptibility profile.

Two-way analysis of variance revealed that the susceptibility of *K. pneumoniae* to different antimicrobials was directly linked to geographic region (*P* <0.001) (Figure 2). By estimating the marginal means, a statistical significance was observed in the means of the proportion of antimicrobial susceptibilities in N. and S. India (*P* <0.001). The log of gross domestic product had an impact on the proportion of susceptibility in India (*P* <0.001), although population density per square km had no significant effect (Supplementary Table 3). In the inter-regional comparison, fosfomycin, imipenem, meropenem, and piperacillin-tazobactam revealed higher means than did the other antimicrobials. In W. India, amikacin showed the highest mean susceptibility, although this difference was not statistically significant (*P* = 0.22) (Table 2).

**Table 2:** Association of antimicrobial agents and regions on the mean (minimum, maximum) proportion of susceptible *K. pneumoniae*.

	North N = 95	South N = 50	East N = 11	West N = 21	*P*-value
Nitrofurantoin	41.18 (16-78) N = 11	48 (23-64) N = 4	24 (—) N = 1	38 (19-57) N = 2	0.744
Fosfomycin	89 (80-98) N = 4	83 (–) N = 1	—	—-	—
Trimethoprim	40.13 (34-56) N = 8	68.2 (58-77) N = 5	72 (—) N = 1	52 (48-56) N = 2	**0.009**
Cefotaxime	37.25 (22-49) N = 4	63 (59-67) N = 2	48 (—) N = 1	71 (—) N = 1	0.14
Ceftriaxone	36.71 (22-49) N = 7	60.5 (52-73) N = 4	48 (—) N = 1	61.5 (47-76) N = 2	**0.031**
Gentamicin	56.5 (37-100) N = 8	78.6 (70-87) N = 5	73 (—) N = 1	77 (73-81) N = 2	0.128
Amikacin	62.8 (39-90) N = 10	85.5 (78-91) N = 4	82 (—) N = 1	84.5 (79-90) N = 2	0.077
Ciprofloxacin	39 (15-61) N = 9	46.2 (38-61) N = 5	69 (—) N = 1	61 (36-86) N = 2	0.313
Amoxicillin	50.23 (23-65) N = 7	68.2 (55-82) N = 5	35 (—) N = 1	65 (63-67) N = 2	0.099
Piperacillin	71.75 (68-79) N = 8	81.2 (77-87) N = 5	65 (—) N = 1	74.5 (74-75) N = 2	**0.016**
Imipenem	72.76 (30-84) N = 9	87.4 (86-90) N = 5	85 (—) N = 1	84 (78-90) N = 2	**0.021**
Meropenem	72.20 (30-87) N = 10	86.4 (85-87) N = 5	83 (—) N = 1	81 (76-86) N = 2	**0.034**
*P*-value	<0.001	< 0.001	—	0.22	

On comparing the mean susceptibilities to different antimicrobials in N., E., W., and S. India, statistically significant differences were observed for trimethoprim-sulfamethoxazole, ceftriaxone, piperacillin-tazobactam, imipenem, and meropenem (*P* <0.05). Trimethoprim-sulphamethoxazole, piperacillin-tazobactam, imipenem, and meropenem showed the highest mean of susceptibilities in S. India compared with the other regions. N. India had the lowest mean susceptibility to ceftriaxone compared with the other regions (Table 2).

**Figure 3:**
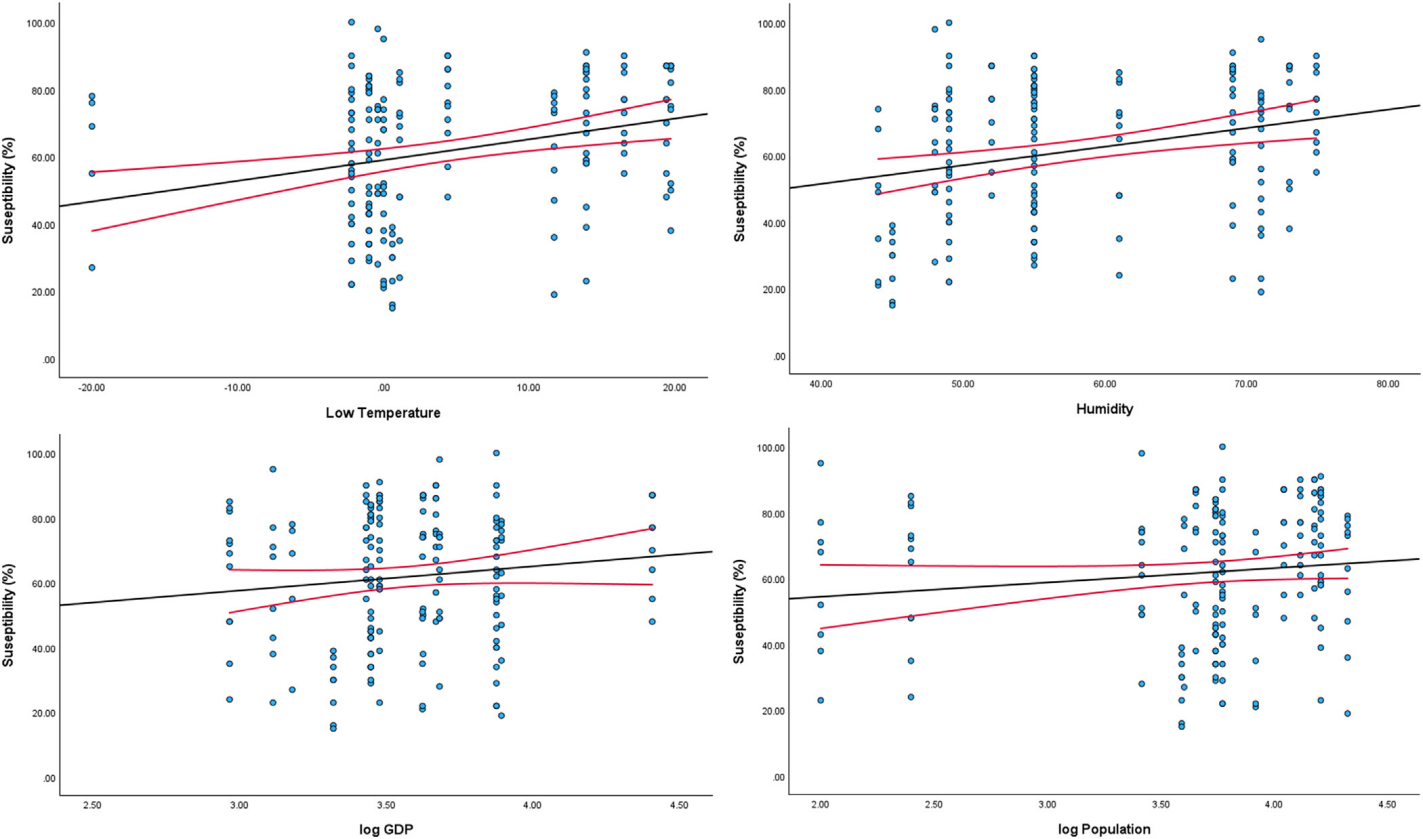
Impact of climatic and socio-economic variables on *K. pneumoniae* antimicrobial susceptibility in India. GDP, gross domestic product.

Humidity, and low and high temperatures (*P* <0.05) had statistically significant effects on the proportion of susceptible *K. pneumoniae* as shown in Figure 3.

## Discussion

The rapid proliferation of ESBLs, AmpC enzymes, and carbapenemases in *K. pneumoniae* complicates the treatment of even simple cystitis [9,10]. The regional variations in India in community UTIs as observed in our study suggest that local antibiograms are a useful tool for promoting antimicrobial stewardship [[11], [12], [13]].

In this study, we tracked the antimicrobial susceptibility of *K. pneumoniae* in patients attending outpatient departments in 18 centers across India. N. India, with its centers located in Delhi, Lucknow, Aligarh, and Patna, showed higher resistance than did the other regions. This region is characterized by a semi-tropical climate, with temperatures increasing from March to July, followed by heavy monsoons over the next 3 months. The low standard of living among large swathes of the population, with concomitant poor access to health care, and substandard or spurious drugs exacerbate the problem of multidrug resistance [14]. In our study, log gross domestic product significantly affected the proportion of susceptibility among *K. pneumoniae* isolates from patients with simple cystitis in India (*P* <0.001). Interestingly, the proportion of susceptible *K. pneumoniae* was directly linked to geographic region (*P* <0.001). Similarly, humidity, and low and high temperatures (*P* <0.05) had statistically significant effects on the proportion of susceptible *K. pneumoniae*. Surprisingly, however, the population density per square km had no significant effect.

Nitrofurantoin showed very low susceptibility against *K. pneumoniae* (39%) throughout India. Susceptibility in N. India (50%) and S. India (48%) was significantly greater (*P* <0.05) than that in W. or E. India or in the Delhi–NCR region. The sites with the lowest susceptibility rates were higher tertiary centers, which received more referrals and treated patients with repeated exposure to antibiotics. Although moderately inhibitory, nitrofurantoin is often prescribed for uncomplicated *K. pneumoniae* cystitis; however, mounting resistance makes it an unreliable drug [15]. ESBL-producing *K. pneumoniae* have a significantly decreased susceptibility to nitrofurantoin compared with non-ESBL producers [16]. The prevalence of ESBL in our study (49%) mirrored the nitrofurantoin susceptibility (39%).

Resistance rates to oral antibiotics were high, suggesting that their empirical use may lead to frequent failures. Co-trimoxazole retained its activity against only 54% of the isolates. Bhargava et al. [17] in 2022 reported an even lower susceptibility of 39.8%, and Vijayganapathy et al. [18] reported a 24% susceptibility in 2021.

The Indian susceptibility to ciprofloxacin (52%) was not significantly lower than that in Europe (57.9%) [19]. The rates for ciprofloxacin ranged from 29-55% in N. India, 36-86% in W. India, 38-61% in S. India, and 22-61% in Delhi, indicating few clear regional differences despite considerable site-to-site differences within regions. In addition to their low susceptibility, safety concerns, and propensity to cause collateral damage, fluoroquinolones are unsuitable for the treatment of simple UTIs.

In such a grim situation, the 89% overall susceptibility to fosfomycin was significantly greater in S. India than in N. India (*P* <0.05). Mohanty et al. [20] reported 91.3% susceptibility. Surprisingly, lower susceptibility rates have been reported in Europe (75.5%) [19]. Fosfomycin is increasingly used to treat UTI caused by resistant non-*E.coli* urinary pathogens. The clinical cure may be affected by a single dose of fosfomycin in healthy individuals with immunocompetence. Studies have reported the efficacy of multiple doses in the treatment of both uncomplicated and complicated UTI caused by multidrug-resistant organisms [21,22]. However, the CLSI and European Committee on Antimicrobial Susceptibility have defined oral fosfomycin breakpoints exclusively for *E. coli*, and breakpoints for any non-*E. coli* Enterobacterales must be extrapolated, a process that is currently unsupported [8,23,24].

The national susceptibility rates of cefepime to third-generation cephalosporins were 51% and 60%, respectively. Our study revealed that the overall prevalence of ESBL-producing *K. pneumoniae* was 49%. The lowest prevalence was observed in W. India (36%), and the highest was 61% in Delhi. Paul et al. [25] reported an ESBL prevalence rate of 26.2% in Assam (N.E. India), whereas Behera et al. [26] reported a combined prevalence of 43% for E. coli and K. pneumoniae from community UTIs in E. India. In 2022, Mohapatra et al. [9] reported an ESBL prevalence of > 50% across four centers.

Among beta-lactam/beta-lactamase inhibitors, lower piperacillin/tazobactam susceptibility (74%) indicated high AmpC prevalence. The highest susceptibility was observed in S., W., and E. India, whereas the lowest susceptibility was observed in N. India *(P* <0.05). Mohapatra *et al.* [9] reported a similar susceptibility (75.1 %). The low susceptibility to amoxicillin/clavulanic acid (46%) may indicate a high prevalence of inhibitor-resistant TEMs. High doses of oral amoxicillin and clavulanic acid have been used to manage ESBL-producing *K. pneumoniae* in Poland [27]. The study participants began with a dose of 2875 g of amoxicillin and 125 mg of clavulanic acid twice daily, with a subsequent reduction in dose every 7-14 days. Prophylaxis continued with 250/125 mg for 3 months [28]. Interestingly, in contrast to *E. coli,* rates of simple cystitis and ciprofloxacin (52%), co-trimoxazole (54%), and amoxicillin susceptibility (55%) were comparable in *K. pneumoniae* [6]. Although Critchley et al. [29] reported higher susceptibilities to these drugs, Hrbacek et al. [30] reported comparable susceptibilities. Although fosfomycin, imipenem, meropenem, and piperacillin-tazobactam indicated higher mean activities than the other antimicrobials, statistically significant differences were observed for trimethoprim-sulfamethoxazole, ceftriaxone, piperacillin-tazobactam, imipenem, and meropenem among the studied regions (*P* <0.05).

Nationwide carbapenem susceptibility was 81.5%, which raises concerns regarding the community-borne dissemination of New Delhi metallo-β-lactamase carbapenemases in *K. pneumoniae* in India. Vijayganapathy et al. [18] reported 99% susceptibility in southern India, whereas Nair et al. [31] reported 87.8% susceptibility in western India, in a four-center study.

## Conclusion

Increasing antimicrobial resistance has made *K. pneumoniae* a challenging uropathogen to treat. This study advocates the creation and dissemination of local antibiograms and promotes evidence-based medicine. A significant difference was observed between the means of the proportions of antimicrobial susceptibility in N. and S. India (*P* <0.001). With low susceptibility to co-trimoxazole and amoxicillin-clavulanic acid, and intrinsically low susceptibility to nitrofurantoin, oral options are rapidly decreasing. The susceptibility to fosfomycin was the highest. Empirically, amoxicillin-clavulanic acid and co-trimoxazole are better choices than fluoroquinolones and third-generation oral cephalosporins. Although single-dose fosfomycin may be effective in healthy individuals with immunocompetence, multiple doses are required in patients with complicated UTI. Multidrug-resistant AmpC isolates may be treated with aminoglycosides, piperacillin-tazobactam, or a combination of both. We advocate that carbapenems be reserved for severe cases.

## Declaration of competing interest

The authors declare that they have no known competing financial interests or personal relationships that could have appeared to influence the work reported in this paper.

## Appendix Supplementary materials

Image, application 1

Image, application 2

Image, application 3

Image, application 4

Image, application 5
