# Supplementary material for: Klebsiella pneumoniae urinary tract infection: A multicentric study highlights significant regional variations in antimicrobial susceptibility across India
Source: IJID Reg. 2025 Feb 19;14:100605. doi: 10.1016/j.ijregi.2025.100605 (PMC11932862; doi:10.1016/j.ijregi.2025.100605)
Supplement: Supplementary file 1 [file mmc1.docx]

**Supplementary Figure 1: Average susceptibility of *Klebsiella pneumoniae* to five major antimicrobial groups**

Third-generation cephalosporins: average of ceftazidime and ceftriaxone

β-Lactam-β-lactamase inhibitors: average of piperacillin-tazobactam

Fluoroquinolones: average of Ciprofloxacin

Carbapenems: average of imipenem and meropenem.
