# Supplementary material for: Klebsiella pneumoniae urinary tract infection: A multicentric study highlights significant regional variations in antimicrobial susceptibility across India
Source: IJID Reg. 2025 Feb 19;14:100605. doi: 10.1016/j.ijregi.2025.100605 (PMC11932862; doi:10.1016/j.ijregi.2025.100605)
Supplement: Supplementary file 2 [file mmc2.docx]

**Supplementary Figure 2: Estimated regional prevalence of ESBLs and carbapenem resistance in *Klebsiella pneumoniae.***
