# Supplementary material for: Klebsiella pneumoniae urinary tract infection: A multicentric study highlights significant regional variations in antimicrobial susceptibility across India
Source: IJID Reg. 2025 Feb 19;14:100605. doi: 10.1016/j.ijregi.2025.100605 (PMC11932862; doi:10.1016/j.ijregi.2025.100605)
Supplement: Supplementary file 3 [file mmc3.docx]

**Supplementary Table 1: Regional Susceptibility profile of *Klebsiella pneumoniae* isolated from community acquired UTI**

| Region | Number of Centers |  | Nitrofurantoin | Fosfomycin | Trimethoprim-Sulfamethoxazole | Cefuroxime | Ceftazidime | Cefotaxime | Ceftriaxone | Cefixime | Cefepime | Gentamicin | Amikacin | Ciprofloxacin | Amoxicillin-Clavulanic acid | Piperacillin-Tazobactam | Cefoperazone/ sulbactam | Imipenem | Meropenem |
| --- | --- | --- | --- | --- | --- | --- | --- | --- | --- | --- | --- | --- | --- | --- | --- | --- | --- | --- | --- |
| **SOUTH INDIA** | **4** | Average | 46% | 83% | 68% | 33% | 60% | 63% | 61% | 52% | 62% | 79% | 86% | 46% | 69% | 82% | 81% | 88% | 86% |
|  |  | Range | 23-64% | 83-83% | 58-77% | 15-48% | 60-60% | 59-67% | 52-73% | 50-53% | 58-68% | 70-87% | 78-91% | 38-61% | 55-82% | 77-87% | 79-82% | 86-90% | 85-87% |
|  |  | Harmonic mean | 38% | 83% | 67% | 26% | 60% | 63% | 60% | 51% | 62% | 78% | 85% | 44% | 68% | 82% | 80% | 87% | 86% |
| **NORTH INDIA** | **5** | Average | 50% | 86% | 37% | 25% | 43% | 38% | 37% | 35% | 39% | 45% | 61% | 37% | 53% | 72% |  | 81% | 77% |
|  |  | Range | 23-78% | 80-95% | 34-43% | 18-31% | 42-43% | 38-38% | 27-43% | 35-35% | 36-43% | 43-49% | 45-84% | 29-55% | 43-65% | 68-79% |  | 77-84% | 71-81% |
|  |  | Harmonic mean | 43% | 86% | 37% | 24% | 42% | 38% | 35% | 35% | 38% | 45% | 57% | 35% | 52% | 72% |  | 81% | 77% |
| **WEST INDIA** | **2** | Average | 38% |  | 52% | 48% | 67% | 71% | 62% | 52% | 74% | 77% | 85% | 61% | 65% | 75% | 51% | 84% | 81% |
|  |  | Range | 19-57% |  | 48-56% | 33-62% | 67-67% | 71-71% | 47-76% | 52-52% | 71-76% | 73-81% | 79-90% | 36-86% | 63-67% | 74-75% | 51-51% | 78-90% | 76-86% |
|  |  | Harmonic mean | 29% |  | 52% | 43% | 67% | 71% | 58% | 52% | 73% | 77% | 84% | 51% | 65% | 74% | 51% | 84% | 81% |
| **EAST INDIA** | **1** | Average | 24% |  | 72% | 59% | 44% | 48% |  |  | 72% | 73% | 82% | 69% | 35% | 65% | 67% | 85% | 83% |
|  |  | Range | 24-24% |  | 72-72% | 59-59% | 44-44% | 48-48% |  |  | 72-72% | 73-73% | 82-82% | 69-69% | 35-35% | 65-65% | 67-67% | 85-85% | 83-83% |
|  |  | Harmonic mean | 24% |  | 72% | 59% | 44% | 48% |  |  | 72% | 73% | 82% | 69% | 35% | 65% | 67% | 85% | 83% |
| **DELHI INDIA** | **6** | Average | 37% | 98% | 44% |  | 46% | 37% |  |  | 55% | 70% | 69% | 47% | 57% | 71% | 45% | 75% | 76% |
|  |  | Range | 21-62% | 98-98% | 34-56% |  | 40-52% | 22-49% |  |  | 55-55% | 49-81% | 51-86% | 22-61% | 42-64% | 68-74% | 45-45% | 68-80% | 71-87% |
|  |  | Harmonic mean | 32% | 98% | 42% |  | 45% | 33% |  |  | 55% | 65% | 66% | 40% | 54% | 71% | 45% | 75% | 76% |
