# Supplementary material for: Klebsiella pneumoniae urinary tract infection: A multicentric study highlights significant regional variations in antimicrobial susceptibility across India
Source: IJID Reg. 2025 Feb 19;14:100605. doi: 10.1016/j.ijregi.2025.100605 (PMC11932862; doi:10.1016/j.ijregi.2025.100605)
Supplement: Supplementary file 4 [file mmc4.docx]

**Supplementary Table 2: Latitude and longitude, temperature, humidity, gross domestic product, population density of the participating centres**

| City | Centers | Latitude | Longitude | Temperature °C | | Humidity  Average  [relative humidity](https://en.wikipedia.org/wiki/Relative_humidity)  (%) | GDP Per capita income  USD | Population density |
| --- | --- | --- | --- | --- | --- | --- | --- | --- |
|  |  |  |  | Average High | Average  Low |  |  |  |
| New Delhi | 4 | [28°36′50″N](https://geohack.toolforge.org/geohack.php?pagename=New_Delhi&params=28_36_50_N_77_12_32_E_type:city_region:IN-DL) | 77°12′32″E | 38 | 7 | 49 | 7,500 | 5,900/km^2^ |
| Gurugram, Haryana | 1 | [28°27′22″N](https://geohack.toolforge.org/geohack.php?pagename=Gurgaon&params=28.456_N_77.029_E_type:city(876969)_region:IN-HR) | 77°01′44″E | 40 | 7 | 48 | 4,800 | 2,600/km^2^ |
| Chandigarh | 1 | [30°45′N](https://geohack.toolforge.org/geohack.php?pagename=Chandigarh&params=30.75_N_76.78_E_type:city_region:IN) | 76°47′E | 37 | 6 | 44 | 4,200 | 9,262/km^2^ |
| Patna, Bihar | 1 | [25.5940343°N](https://geohack.toolforge.org/geohack.php?pagename=Patna&params=25.5940343_N_85.1375530_E_type:city_region:IN-BR) | 85.1375530°E | 36.6 | 8 | 61 | 925 | 250 km^2^ |
| Mumbai, Maharashtra | 1 | 19°04′34″N | 72°52′39″E | 32 | 18 | 71 | 7,800 | 21,000 km^2^ |
| Surat, Gujarat | 1 | [21°12′18″N](https://geohack.toolforge.org/geohack.php?pagename=Surat&params=21_12_18_N_72_50_24_E_type:city(6936534)_region:IN-GJ) | 72°50′24″E | 35 | 20 | 55 | 4,665 | 15,000/km^2^ |
| Pondicherry | 1 | [11°55′N](https://geohack.toolforge.org/geohack.php?pagename=Pondicherry&params=11_55_N_79_49_E_type:city(244377)_region:IN-PY) | 79°49′E | 30 | 25 | 74.9 | 2,700 | 13,000/km^2^ |
| Thiruvananthapuram, Kerala | 1 | [08°31′26.8″N](https://geohack.toolforge.org/geohack.php?pagename=Thiruvananthapuram&params=08_31_26.8_N_76_56_11.8_E_type:city_region:IN) | 76°56′11.8″E | 32 | 23 | 73 | 4,219 | 4,500/km^2^ |
| Chennai, Tamil Nadu | 2 | [13°4′57″N](https://geohack.toolforge.org/geohack.php?pagename=Chennai&params=13_4_57_N_80_16_30_E_region:IN-TN_type:city(6748026)) | 80°16′30″E | 36 | 28 | 69 | 3000 | 16,000/km^2^ |
| Lucknow, UP | 3 | [26°51′N](https://geohack.toolforge.org/geohack.php?pagename=Lucknow&params=26_51_N_80_57_E_type:city_region:IN-UP) | 80°57′E | 40.5 | 8 | 55 | 2, 800 | 5,500/km^2^ |
| Srinagar, Jammu & Kashmir | 1 | [34°5′24″N](https://geohack.toolforge.org/geohack.php?pagename=Srinagar&params=34_5_24_N_74_47_24_E_type:city(1180570)_region:IN) | 74°47′24″E | 21.3 | 0.3 | 55 | 1512 | 4,000/km^2^ |
| Aligarh | 1 | [27.88°N](https://geohack.toolforge.org/geohack.php?pagename=Aligarh&params=27.88_N_78.08_E_type:city(874408)_region:IN-UP) | 78.08°E | 40.5 | 7 | 71 | 1,300 | 100/km^2^ |
