# Supplementary material for: Klebsiella pneumoniae urinary tract infection: A multicentric study highlights significant regional variations in antimicrobial susceptibility across India
Source: IJID Reg. 2025 Feb 19;14:100605. doi: 10.1016/j.ijregi.2025.100605 (PMC11932862; doi:10.1016/j.ijregi.2025.100605)
Supplement: Supplementary file 5 [file mmc5.docx]

| **Supplementary Table 3. The result of two-way ANOVA for the proportion of susceptible *K. pneumoniae* based on the type of antimicrobial drug and geographic regions by adjusting regional covariates (low/high temperature, humidity, log GDP and log density of population (per km)** | | | | | | |
| --- | --- | --- | --- | --- | --- | --- |
| Variables | Sum of Squares (SS) | df | Mean Square (MS) | F | P-value | Effect size |
| Drug | 37204.122 | 11 | 3382.193 | 21.371 | **<.001** | .600 |
| Region | 6971.148 | 3 | 2323.716 | 14.683 | **<.001** | .219 |
| High temp | 1079.463 | 1 | 1079.463 | 6.821 | **.010** | .042 |
| Low Temp | 4358.349 | 1 | 4358.349 | 27.539 | **<.001** | .149 |
| Humidity | 790.027 | 1 | 790.027 | 4.992 | **.027** | .031 |
| Log GDP | 3608.218 | 1 | 3608.218 | 22.799 | **<.001** | .127 |
| Log Population | 496.756 | 1 | 496.756 | 3.139 | .078 | .020 |
